# Does socioeconomic position and gender affect human-nature interactions?

**DOI:** 10.1007/s10389-022-01762-8

**Published:** 2022-10-18

**Authors:** Ximena Tiscareno-Osorno, Jihad Hossaini, Sophia Chabursky, Nesma Sayed, Ilayda Temizarabaci, Wiebke Hähl, Jürgen Beckmann

**Affiliations:** 1 Department of Sport and Health Sciences, Technical University of Munich, Georg-Brauchle-Ring 60_62/IV., 80992 München, Germany; 2 School of Human Movement and Nutrition Sciences, University of Queensland, Brisbane, Australia

**Keywords:** Human–nature interactions, Nature connectedness, Commitment to the environment, Mental health, Socioeconomic position, Gender

## Abstract

**Aim:**

The growing number of mental health problems worldwide is alarming. Encouraging human–nature interactions (HNIs) could help to tackle this issue. For this reason, the aim of the present research was to investigate certain components that promote HNI in two groups of students with different socioeconomic positions (SEPs) in Mexico. HNIs describe the direct relationship between humans and nature. HNIs are composed of elements such as connectedness to nature (CN) and commitment to the environment (CE), and are beneficial to both physical and mental health. However, the impact of CN and CE on people’s lives seems to depend on their SEP, which has been investigated mainly in developed countries where SEP is less salient compared with developing countries.

**Methods:**

A survey was sent to students from two universities representing each group. A total of 210 surveys was collected.

**Results:**

The results showed no differences in CN (*p* = .480) and CE (*p* = .421) regarding SEP. However, gender differences were found with females from a low SEP showing higher levels of CN than men (*p* = .015) from the same SEP. For high SEP, no differences were found. In addition, men showed a higher CE than women, regardless of their SEP.

**Conclusion:**

Given the high vulnerability of women to mental health problems, it is of major importance to conduct more research considering the relationship between gender, HNI, SEP, and health in developing countries.

The Earth is what we all have in common

***Wendell Berry***

Global mental health issues are increasing alarmingly (OECD 2018; World Health Organization 2021c). According to the World Health Organization (WHO), a 13% increase in mental diseases was detected up to 2017, and about 20% of children and adolescents worldwide have a mental health condition (World Health Organization 2019). Therefore, several organizations have begun to promote alternatives to improve mental health and well-being. Various schemes have been proposed to target the increase of human–nature interactions (HNIs) (OECD 2021; United Nations 2021). HNI is an approach that aims to increase the interaction between human beings and their environment, and has been shown to have multiple benefits, with the most important being its positive effects on mental health (Seymour 2016). The effects of HNI can be explained by the biophilia hypothesis, which is the most common theory in environmental psychology (Davis et al. 2009). This hypothesis states that humans have a special relationship with nature and a need to connect with it (Kellert and Wilson 1993). Current research has linked the hypothesis to the understanding of HNI, which involves the effects of HNI on physical and mental health as key components (Brymer et al. 2019; Zuo et al. 2015). Accordingly, greater contact with nature has been shown to increase well-being (Wolsko and Lindberg 2013; Zuo et al. 2015), and accessibility and exposure to nature has been shown to be associated with the prevention of physical and mental illnesses (Cox et al. 2017). As suggested by the biophilia hypothesis, this might be due to a positive emotional state evoked by nature (Lumber et al. 2017). This positive state has not only been observed on a subjective level but also within the brain. In an EEG study on brain activity and nature, Mahamane et al. (2020) analyzed the event-related potential of a group of people exposed to both natural spaces and built up areas. Partcipants showed a higher late positive potential, a marker of emotional dysregulation, when exposed to nature than when exposed to urban settings. This is indicative of a more positive emotional state during the viewing of nature and suggests that natural spaces are perceived as more pleasurable. However, there remains a lack of understanding regarding the exact psychological mechanisms behind the association between HNI and mental health (Brymer et al. 2019; Zuo et al. 2015).

Two constructs that are highly related to HNI and used in research are connectedness to nature (CN) (Brymer et al. 2019; Mayer et al. 2008) and commitment to the environment (CE) (Davis et al. 2009; Seymour 2016). On the one hand, CN is considered as the means by which people make nature part of their self-representation (Schultz 2002). A high CN has positive effects on happiness (Capaldi et al. 2015; Leavell et al. 2019; Richardson and McEwan 2018), quality of life (Cervinka et al. 2012), and well-being (Capaldi et al. 2014; Howell et al. 2012). On the other hand, CE refers to the psychological attachment of a person to nature (Davis et al. 2009). Accordingly, CE is a predictor of past and future behavior toward the environment (Coy et al. 2013; Davis et al. 2009, 2011, 2015) and therefore has a strong relationship with pro-environmental behavior (Davis et al. 2015). For this reason, it can be associated with attention restoration, a reduction in mental fatigue, and an improvement in cognitive functioning (Annerstedt van den Bosch and Depledge 2015; Davis et al. 2009, 2011). Overall, research has shown that these two constructs are strongly related to each other (Yu et al. 2019).

Up until the present moment, it remains unclear how universal the relationship between mental health and CN, as well as CE, is. One major question, for example, is whether the relationship is independent of external factors, such as socioeconomic position (SEP). SEP is considered a broad construct that reflects not only the financial but also the social and cultural condition of an individual, which increases the complexity of its measurement (Cowan et al. 2012). However, SEP can be highly relevant when it comes to HNI. For example, socioeconomic inequalities have been shown to be associated with the relationship between exposure to nature and mental health as well as well-being (Mitchell et al. 2015). This is important to consider because mental health and well-being also vary depending on SEP. For example, people with higher incomes are able to live in neighborhoods where they can access more services, as well as finance higher costs, to cover their health needs (Marmot 2017). At the same time, people with a higher income may have greater accessibility to nature (Astell-Burt et al. 2014; Mears et al. 2019), which is also related to health (Annerstedt van den Bosch and Depledge 2015). Considering that accessibility to nature highly depends on SEP, it could be assumed the positive effects of HNI on mental health might also depend on SEP.

SEP varies greatly both between and within countries, with differences being very pronounced between developing and developed countries. However, to date, most of the research has been conducted in developed countries and only a few researchers have assessed this relationship in developing countries. For example, Scopelliti et al. (2016) examined CN between the different income groups in Colombia. They found that people belonging to the middle-income group presented a higher well-being as well as a higher CN. Similarly, a study conducted in Chile revealed that the lowest socioeconomic group scored the lowest in pro-environmental behavior (Bronfman et al. 2015). While these findings highlight that a low SEP is associated with low HNI, further research suggests that in low-income countries, SEP is only weakly related to environmental concerns, as opposed to high-income countries, where it is strongly related (Pampel 2014). These findings suggest that SEP plays a particular role in developing countries in regard to CN, CE, and ultimately, in health.

In summary, previous literature asserts that CN and CE are associated with mental health and at the same time are linked to SEP. However, the impact of CN and CE has been mainly studied in developed countries where SEP and the social gradient are less pronounced, while there is a scarcity of research in developing countries. Existing research shows that a low SEP is associated with reduced HNI. For this reason, the present study investigates CN and CE and their associations with SEP in a developing country, namely Mexico. We hypothesize that the reported CN and CE differ in individuals with high and low SEP.

## Methods and materials

This cross-sectional survey assessed HNI in Mexican students enrolled in a health-related programs at a public and private university. Both universities were chosen because of the difference in the semester fee. While public universities may be free, or have a very low cost, because they are subsidized by the government, private universities have high fees. Research shows the SEP of students is usually a reflection of the access that the family has to financial, cultural, social, capital, and human resources. Thus, in general, it is a reflection of the SEP of the family household (OECD 2019). Furthermore, students attend different institutions according to their SEP. These institutions differ not only in their facilities but also in their teaching, the experience or updating of their teachers, and the access and relationship that students and even parents can have with them (Cowan et al. 2012). Finally, it has been observed that students with low SEP are more likely to attend institutions where they do not have easy access to social capital, which is related to networks that later help them to obtain personnel or professional gains (Coleman 1988).

### Participants

A total of 349 students agreed to take part in the survey, and 289 of the respondents completed it. After removing respondents with missing data, 210 students were finally included in the study. Undergraduate students of the Instituto Tecnológico y de Estudios Superiores de Moneterey - Campus Ciudad de Mexico (Mexico City; private university; *n* = 106) and Universidad Autónoma Metropolitana - Unidad Xochimilco (Mexico City; public university; *n* = 104) enrolled in a health related program (i.e., medicine, nutrition, dentistry, psychology) were surveyed.

All students had been residing in Mexico for at least one year. A required sample size of 67 participants per group was calculated using G*Power Version 3.1 (Faul et al. 2009), assuming a medium effect size of 0.5 (Cohen 1977) for a two-tailed Wilcoxon–Mann–Whitney test with an α-error probability of 5% and a power of 80%.

### Procedure

After giving informed consent, the participants were asked to provide demographic information regarding gender, age, nationality, education level, household expenditure, university type (public or private), occupation, and time residing in Mexico City. CN and CE were then assessed using two validated questionnaires. The Spanish version (Pasca et al. 2017) of the Connectedness to Nature Scale (CNS) (Mayer and Frantz 2004) was used to assess CN. This scale is commonly used to measure an individual’s trait of feeling connected to the natural word in an affective and experiential manner (Mayer and Frantz 2004; Pasca et al. 2017). The Spanish adaptation of the CNS is composed of seven items that ask for different experiences with nature (e.g., *“I often feel at one with the natural world around me”)* with responses indicated on a five-point Likert scale ranging from “1 – strongly disagree” to “5 – strongly agree.” The Spanish scale utilized was adapted to Mexican Spanish by a panel of three experts familiar with the terminology and topic following the guidelines of the World Health Organization (2021a). This adaptation was required to prevent conceptual confusion based on culture-specific language differences. The adaptation was tested with a sample of 49 students. Reliability testing revealed a good result as indicated by a Cronbach’s α of .87. The reliability achieved in the final sample showed a Cronbach’s α of .83.

The Commitment to the Environment Scale (CES) was developed by Davis et al. (2009) and is commonly used to assess psychological attachment toward the natural environment. It consists of 11 items that ask for the extent to which individuals agree with various attitudes about their relationship with nature (e.g., “*I believe that the well-being of the natural environment can affect my own well-being*”). Responses range from “0 – do not agree at all” to “8 – agree completely.” Since no validated Spanish version of the CES existed, we therefore translated the English questionnaire to Mexican Spanish. The translation again followed the guidelines of the World Health Organization (2021a); with the translation being done by a group of health professionals familiar with the terminology. After that, the Mexican Spanish scale was discussed by a panel of three experts, who made any changes required to the terminology used. There were no adaptations to the scale. Finally, the resulting scale was translated back to English by an independent translator. The translated scale was initially tested with a sample of 49 students, which showed a very good internal consistency (Cronbach’s α = .93). The reliability achieved in the final sample showed a Cronbach’s α of .77.

## Results

The aim of the present study was to investigate the CN and CE of Mexican students from different SEPs. To test financial differences between the students of the two universities, we compared household expenditure using a two-sample t-test. Results showed that the mean (*M*) household outlay (pesos/month) of public was 4.359 and the standard deviation *(SD)* was 3.851. For private university students the results differed significantly showing an *M* = 3.0571, *SD* = 2.8993, students t distribution of 9.14 with 107 degrees of freedom (t(df)), and a p-value (*p*) less than .000. As expected, household outgoings from students of the private university were higher than those of students from the public university.

Two Mann–Whitney U-tests were performed to test the hypotheses regarding differences in CN and CE between the two universities. This non-parametric test was used based on the non-normal distribution as indicated by D'Agostino normality tests. According to the Mann–Whitney U-tests, there was no significant difference between the groups, neither for CN (*p* = .480) nor CE (*p* = .421) (see Table 1).

**Table 1:** Mann–Whitney U-tests comparing connectedness to nature and commitment to environment of students from two universities

	Public university		Private university					
Human-nature interactions	*Mdn*	M_rank_	*Mdn*	M_rank_	*U*	z	p	r
Connectedness to nature	3.86	53.5	3.93	52.5	5822.5	0.71	.480	.04
Commitment to the environment	3.55	53.5	3.73	52.5	5867.0	0.81	.421	.05

*N* = 210

M_rank_= Mean rank

Mdn= Median

U = U-value from the Mann–Whitney U-test

z = z score

p = *p*-value

r = Pearson’s correlation coefficient

All *p*-values are two tailed

**Fig. 1:**
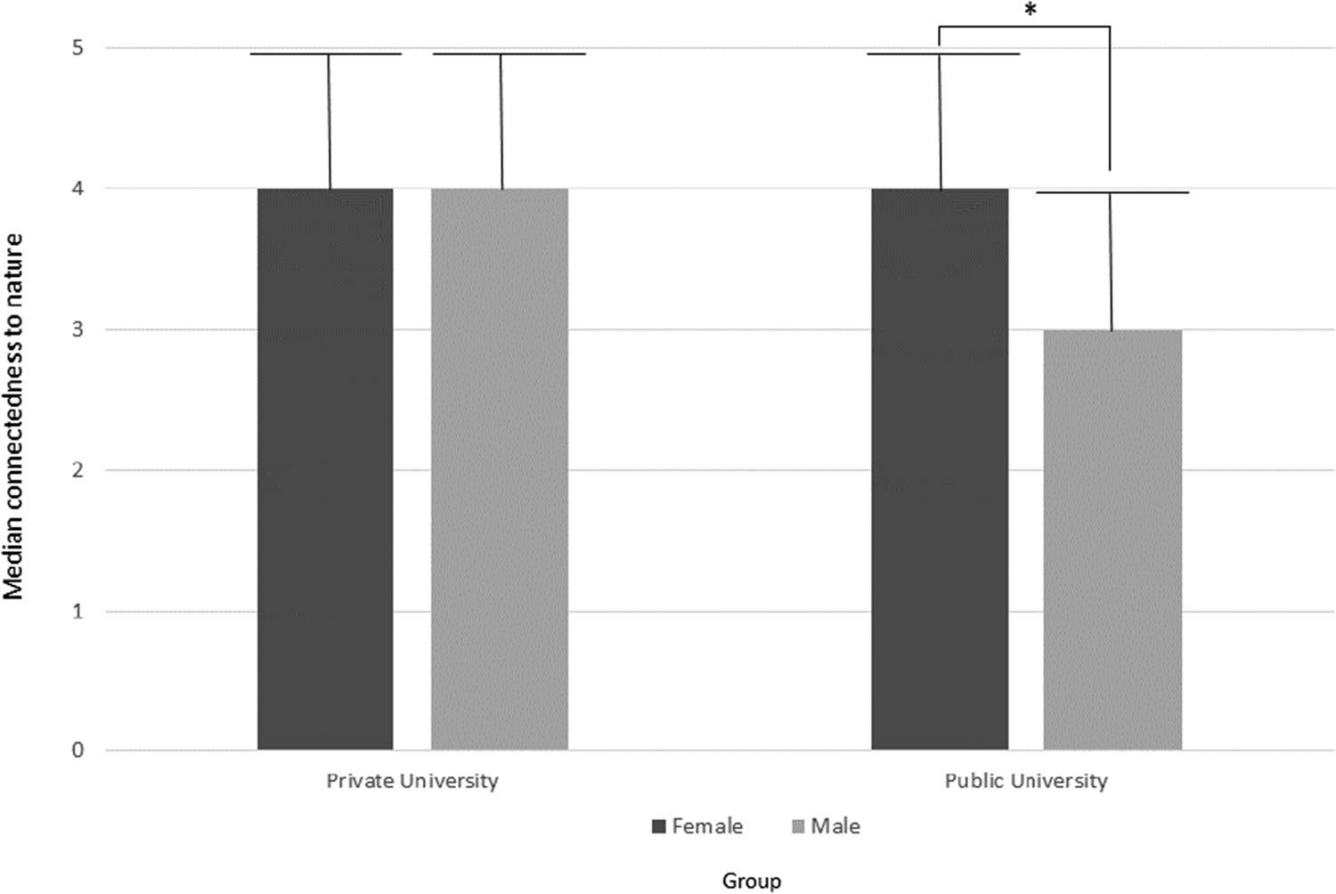
Median connectedness to nature scores grouped by gender and university * = significant difference

An explanatory analysis was conducted to further investigate the role of gender within the relationship of CN and CE with SEP. Due to the low and unequal numbers of partcipants, we decided to compare female (*n*_public_ = 78, *n*_private_ = 73) and male (*n*_public_ = 28, *n*_private_ = 31) students within each institution using a non-parametric test. As can be seen in Fig. 1, a Mann–Whitney U–test comparing male and female students of the two universities revealed a significant difference for the public university, showing a U-value from the Mann–Whitney U-test (*U) of* 753.5, a z score (*z)* of 2.43 , *p* .015, and a Pearson’s correlation coefficient (r) of .16. The median (*Mdn*) of the CN values for male students was 3.57, which was lower compared to female students (*Mdn* = 3.86). For the private university, no differences were found.

**Fig. 2:**
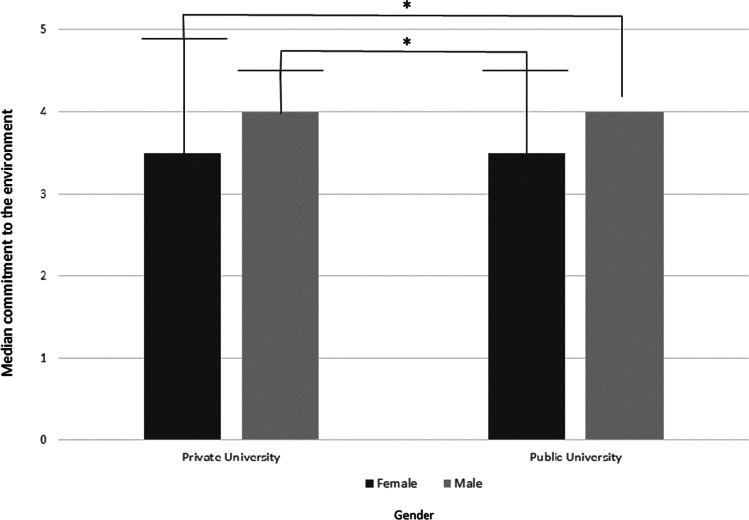
Median commitment to the environment scores grouped by gender and university.* = significant difference

Additionally, there was a significant difference between CE values of men and women, *U* = 3504.5, *z* = 2.4 , *p* = .016, *r* = .17. Male students (*Mdn* = 3.18) showed a higher CE compared with female students (*Mdn* = 3.46) regardless of their university (see Fig. 2).

## Discussion

The present study investigated the relationship between SEP, CN, and CE of students in a developing country. This research was composed of a sample of students from a public and private university in Mexico. The type of university to which the students belonged was used as an indicator of their SEP (Galobardes et al. 2006; Moreno-Maldonado et al. 2018). Our hypothesis was that CN and CE vary between high and low SEP individuals. In contrast to our hypothesis, the results revealed no difference in CN and CE between individuals with high and low SEP. These findings are in line with Iskandar et al. (2017) who found no difference between SEP and environmental concern. They suggested that this could be due to lower levels of environmental concerns in developing countries. This assumption is further supported by Pampel (2014) who showed that the association between SEP and environmental concern is weak in low-income nations, whereas SEP and environmental concern are strongly associated in high-income countries.

However, the present findings also contradict other research that supports an association between HNI and income in developing countries. For example, Scopelliti et al. (2016) investigated the effect of contact with nature on well-being in three groups with different incomes in Colombia. They found that the middle-income group had a higher CN compared to the two other high and low income groups. The researchers supposed that this was due to the fact that the low-income group, as well as the high-income group, related their well-being primarily to their income, as opposed to the middle-income group. Hence, they concluded that income plays an important role in HNI. However, the differentiation between the SEP groups is not mentioned explicitly and might therefore not be comparable to the present study, particularly when considering that the gross domestic product in Colombia is lower than in Mexico. Also, their sample was more heterogeneous since they used an opportunistic sample in a park instead of predefining the target group.

In addition, we found a significant difference regarding gender, CN, and SEP, with female students with a low SEP reporting higher levels of CN than male students with a low SEP. In contrast, these differences were not observed in individuals with a high SEP. This is an important finding, considering that according to the theory of “facultatively-mediated sex differences” (Schmitt 2015), gender differences in personality are related to ecological stress, with less ecological stress leading to stronger differences. Therefore, gender differences in personality are more likely in developed countries (Kaiser et al. 2019). However, this might be counteracted by women having a stronger connection to nature and a greater preference for spending time in outdoor recreational activities than men, regardless of their origin. Moreover, these gender differences have been reported particularly for the present age group, which belongs to the millennial generation (Reese et al. 2020). Furthermore, male students reported higher levels of CE than female students, regardless of their SEP. These findings are in line with Vicente-Molina et al. (2018) who found that men show more pro-environmental behaviors compared with women. This outcome, however, contradicts Clayton et al. (2021) who demonstrated that women are more involved and interested in changing their behavior in favor of the environment than men. However, as they mentioned in their study, their conclusions should be taken with caution, as they may not be considering fundamental factors, such as education.

Finally, considering the increased vulnerability of women to mental disorders (World Health Organization 2021b) and the positive mental health outcomes reported for HNI (Brymer et al. 2019), the observed gender differences are of particular relevance. Therefore, it is important to understand the complex relationship between gender, nature, and health (MacBride-Stewart et al. 2016). In this respect, females are the most vulnerable group due to their sensitivity concerning changes in nature and the environment. This increased susceptibility, potentially indicated through an increased level of CN, might limit the health benefits of HNI in women if the quality of nature is low (MacBride-Stewart et al. 2016). Environmental degradation, often evident in low income areas, particularly within developing countries, might therefore pose a risk to women’s health (MacBride-Stewart et al. 2016; Majeed and Ozturk 2020; Wang and Dong 2019; White et al. 2019). Accordingly, it is essential to include health outcomes in addition to CN to further understand the connection between gender, nature, and health. Future research should focus specifically on health outcomes in women from different SEPs and environmental areas in order to derive practical implications that help foster female participation in pleasant natural environments.

### Limitations

Limitations of the present study should be considered. First, the investigation was conducted during the COVID-19 pandemic. Therefore, the participants were more prone to answer the survey from a different area than Mexico City, for example, some students might have gone to their country houses during lockdown, or simply returned to their hometowns where they were more likely to encounter and interact with nature. Therefore, the results may not represent the general level of CN or CE.

Second, we assumed that the two universities were indicative of different SEP, although we were not able to fully test this assumption, while a comparison of financial conditions (such as household expenditure) revealed a difference between the two universities. Because the *SD* of both groups was quite high, this cannot be seen as strong support. For future research, we suggest using a measure that allows comprehensive assessment of SEP, including social and cultural backgrounds.

Finally, to date, there is no validated questionnaire that measures CN in urban conditions. Therefore, we used a questionnaire designed to measure this construct in a general manner only. It is important to highlight that the use of a scale specialized in measuring CN in cities could enable us to gain a clearer picture of HNI in people living in the metropolis. For instance, the way people in contexts where access to nature is more limited relate to and perceive nature may be different than that of people living in rural places where there are more green spaces. However, in the current study, both universities were located in the same neighborhood within Mexico City. Therefore, access to nature and the environmental setting were comparable.

## Conclusion

In summary, this study shows that students from different SEPs, indicated by two disparate universities, had a very similar CN and CE. However, a significant contrast was found regarding gender and CN depending on SEP, with women with a low SEP reporting higher levels of CN than men with a low SEP. In addition, another significant difference found revealed that men had a higher CE, regardless of their SEP. This suggests the importance of conducting future research that addresses the relationship between gender, nature, and SEP in developing countries to help increase health benefits of HNI.

## Data Availability

To access any of the research materials related to this article (i.e. data), please contact the author listed in the correspondence. The data of the study will be kept for 10 years.
